# Deep learning-based differentiation of FLASH ultrasound windows

**DOI:** 10.1186/s12917-026-06021-6

**Published:** 2026-10-23

**Authors:** Laura Marlene Köninger, Tobias Müller, Stefan Gesell-May, Jessika-M.V. Cavalleri, Silvio Kau-Strebinger, Anna May

**Affiliations:** 1 Equine Hospital, Ludwig-Maximilians-University Munich, Munich, Germany; 2 Centre for Equine Ophthalmology/anirec, Munich, Germany; 3 Equine Internal Medicine, Clinical Centre for Equine Health and Research, University of Veterinary Medicine Vienna, Vienna, Austria; 4 Department of Biological Sciences and Pathobiology, Unit of Morphology, University of Veterinary Medicine Vienna, Vienna, Austria

**Keywords:** Artificial intelligence, Ultrasound, Deep learning, Horse, Teaching, Abdomen

## Abstract

**Background:**

Equine colic emergencies present highly stressful, time-critical scenarios for both clinicians and patients, thus rapid and accurate diagnostic triage is paramount. To support veterinarians under these high-pressure conditions, deep learning tools can be leveraged for automated image recognition to assist in localizing abdominal organs and interpreting FLASH (Fast Localized Abdominal Sonography of Horses) ultrasound windows.

**Objectives:**

A software tool was developed to identify FLASH-specific ultrasound windows on the equine abdomen and to recognize ultrasonographic organ patterns.

**Study design:**

Retrospective-prospective comparison of software and clinical examination, model development.

**Methods:**

A deep learning approach for image classification was used to train software to assign ultrasound images to FLASH windows.

**Results:**

Strongest software performance was observed for the duodenal window, with all test images correctly classified, corresponding to a recall of 1.00. Nephrosplenic window demonstrated high classification accuracy, but 23% of images were misclassified as gastric window. While 84% of ventral images were correctly classified, 16% were predicted as duodenal window. Most pronounced confusion was observed between ventral and thoracic windows, with only 55% of thoracic images correctly identified. Left and right middle third FLASH windows 4 and 6 were substantially underrepresented and were therefore excluded from further analyses. In addition, these windows exhibited high anatomical similarity, which further complicated their differentiation.

**Main limitations:**

Sources of selection bias was interpretation of windows and the definition of normal physiological ultrasound images by the examiners. The specific FLASH windows were not homogeneously dispersed in the dataset (e.g., FLASH windows 4 and 6 were underrepresented).

**Conclusions:**

The developed deep learning tool demonstrates the feasibility of automated recognition of standardized equine FLASH windows and may support ultrasound training as an educational aid for first-day competencies. Future incorporation of organ-level annotations and probe position information may improve discrimination between anatomically similar windows and enhance model performance.

## Background

**Fig. 1:**
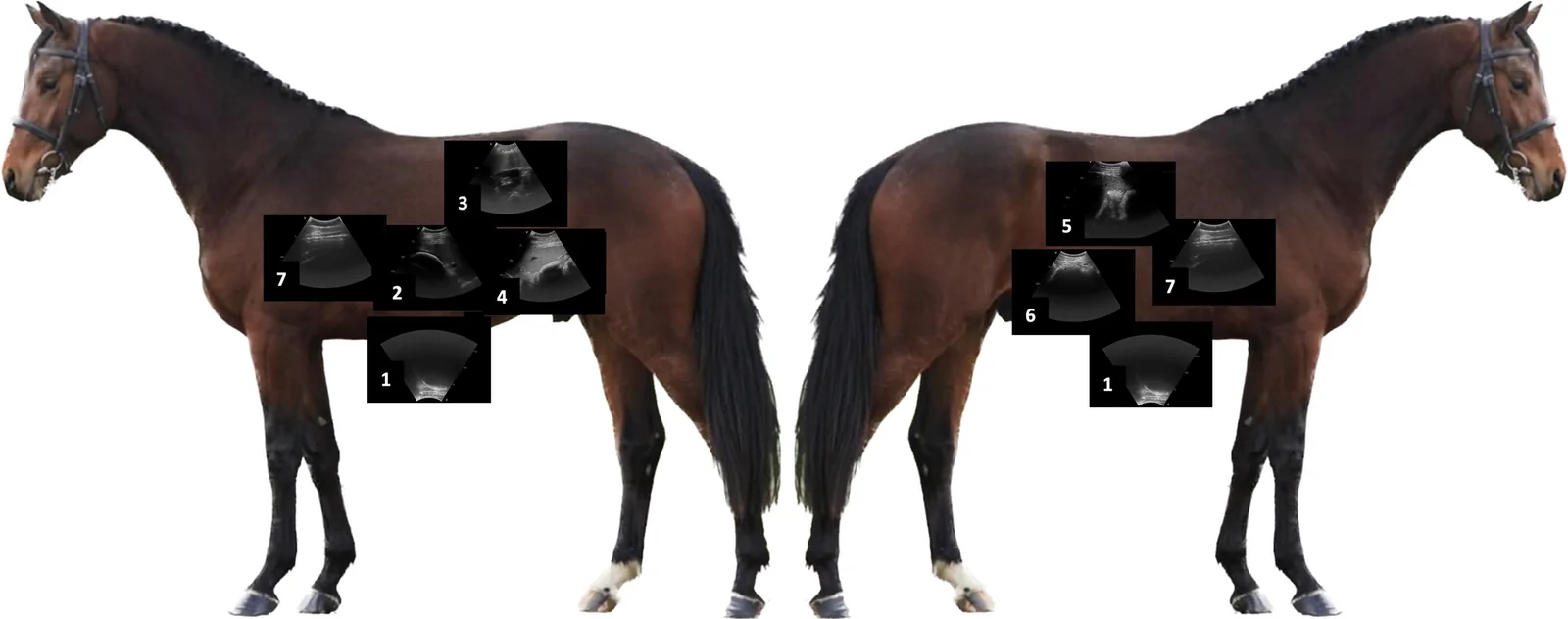
FLASH (Fast Localised Abdominal Sonography of Horses) Windows in the horse and the organs that can physiologically be identified at specific sites. Window 1: Ventral window – large intestine, Window 2: Gastric window – stomach, spleen, Window 3: Nephrosplenic window – kidney, spleen, Window 4: Left middle third – large intestine, spleen, Window 5: Duodenal window – liver, duodenum, large intestine, Window 6: Right middle third – large intestine, Window 7: Cranial ventral thorax – lung

Fast Localised Abdominal Sonography of Horses (FLASH) is a focused point-of-care ultrasound protocol that examines predefined abdominal and thoracic windows to rapidly assess clinically relevant abnormalities in horses with colic and other emergency conditions. The technique can help with decision-making in colic horses and has been shown to be more accurate for detecting lesions requiring surgical intervention than transrectal palpation [1–3] It has therefore become a standard procedure in most equine hospitals to diagnose gastrointestinal disease in emergency settings [4]. The technique comprises a thoracic window, and six abdominal ultrasonographic windows on both sides of the horse where intestinal contents, wall thickness, distension, motility, and presence of free fluid is evaluated as well as presence and size of kidney and stomach (Fig. 1).

In human medicine, application of artificial intelligence (AI) in ultrasound diagnostics started approximately 40 years ago [5] and has advanced since then [6–11]. Medical ultrasound analysis mainly consists of localization, which identifies the position of anatomical structures; segmentation, which delineates their boundaries at the pixel level; classification, which assigns images or regions to predefined categories; and registration, which aligns two or more images to enable comparison of the same anatomical structures [12, 13] – areas which can be supported by AI and more specifically by deep learning. Studies showed that deep learning can be extremely helpful in areas with large amounts of digitized information available, as there is in ultrasound diagnostics [14]. Deep learning can help improve efficiency and accuracy of diagnosis in this field and may reduce human errors because of fast and reliable detection and lesion segmentation. In human medicine, artificial intelligence and specifically deep learning has made vast advances in recognizing patterns in medical ultrasound images and in automatically diagnosing conditions, such as breast cancer [15–21], cardiac disease [22–24], thoracic pathologies [25], thyroid disease [26–32], prostate cancer [33–35], brain disease [36–40], staging fetal development and detecting fetal abnormalities [41–46]. But also in veterinary medicine the field of diagnostic imaging is being transformed by integration of AI tools. While in conventional radiology, computed tomography, and magnetic resonance imaging different applications to various body parts have been highlighted, studies on AI in veterinary ultrasound have been comparably scarce [47–50]. Recent studies developed a deep learning-based tool for ultrasonographic classification of canine chronic kidney disease [51] and canine cataracts [52]. Another study assessed whether deep learning models can be reliably used to interpret ultrasound images obtained from working dogs using the Global FAST exam [53], which is like the FLASH examination in horses [54]. In horses, deep learning has been used in the field of diagnostic ultrasound to show repeatability of measurement of cross-sectional area of the multifidus muscle in the horse [55]. Studies have shown that ultrasound teaching is underrepresented at veterinary schools [56] although being a first-day competency [57] and that inexperienced veterinarians need to practice their ultrasound skills before they can reliably diagnose gastrointestinal disease in horses [58]. The increasing demand for ultrasound proficiency, combined with limited training resources and the need for hands-on supervision, has stimulated interest in simulation-based and computer-assisted educational approaches [59].

Therefore, the aim of this study was to develop an ultrasound tool which can support equine ultrasound teaching at veterinary schools by serving as a supplement to conventional teaching, allowing students to practice basic probe orientation and standardized FLASH window recognition before scanning live horses. This may enable more efficient use of practical teaching time by allowing students to identify the relevant anatomical windows more quickly once they perform examinations on live animals, especially in stressful emergency situations like equine colic. The developed deep learning ultrasound tool was trained to recognize the different FLASH windows and identify the pictured organs in the specific region in healthy horses. Images that were obtained from videos taken in the specific FLASH windows were used for training the tool. We hypothesized that the model would accurately classify the different FLASH windows and identify the corresponding organs in ultrasound images obtained from healthy horses.

## Materials and methods

### Study type and aim

Retrospective-prospective proof-of-concept study using both archived ultrasound examinations and ultrasound examinations prospectively acquired specifically for this study to develop and evaluate a deep learning model for automated classification of FLASH ultrasound windows.

### Animals

Ethical approval was granted by the State of Bavaria, protocol number ROB-55.2-2532.Vet_03-20-49.

Six healthy horses owned by the Ludwig-Maximilians-University, Munich, Germany, were used to obtain ultrasound images (retrospectively collected and prospectively acquired data). The group consisted of 1 Haflinger stallion, 1 Haflinger mare, 1 Warmblood mare, 2 standardbred mares, and 1 thoroughbred mare. The horses’ median age was 19 years (IQR: 10–24 years).

### Image acquisition

The horses’ hair coat was clipped and the skin was cleaned with alcohol, and 70% isopropyl alcohol was used as the sole coupling agent. All examinations were performed using a GE ultrasound machine (Logiq Vivid E95, GE HealthCare, Chicago, IL, USA) equipped with a C1-6 convex array transducer at a frequency of 2.8 MHz. The ultrasound examinations were performed by one resident and one diplomate of the European College of Equine Internal Medicine (ECEIM). Ultrasound images were acquired according to the Fast Localised Abdominal Sonography of Horses (FLASH) protocol described by Busoni et al. (2011) [1]. Initially, seven standard acoustic windows were included in the analysis: FLASH 1 (ventral window), FLASH 2 (gastric window), FLASH 3 (nephrosplenic window), FLASH 4 (left middle window), FLASH 5 (duodenal window), FLASH 6 (right middle window), and FLASH 7 (thoracic window; Table 1). All cine loops were recorded during repeated examinations of the six horses using the standardized FLASH examination, and each recording was assigned to its respective FLASH window at the time of image acquisition based on probe position and anatomical landmarks. Subsequently, individual frames were extracted from the cine loops and subsequently assigned to their respective FLASH window by two ECEIM board-certified examiners. All images were exported in digital format and used for model training and evaluation.

**Table 1:** FLASH windows and their specific features in colic [1]

Window	Main organs/features	Key structures and sonographic assessment
1 Ventral	Free fluid, small intestine	Presence of peritoneal free fluid and small intestinal distension or motility
2 Gastric	Stomach, liver, spleen	Gastric size, gastric wall, and echogenicity of the liver and spleen
3 Nephrosplenic	Spleen, left kidney	Nephrosplenic space and anatomical relationship between spleen and left kidney
4 Left middle third	Left colon	Large colon position, wall thickness, and motility
5 Duodenal	Duodenum	Duodenal diameter, wall thickness, and motility
6 Right middle third	Caecum, Colon	Right-sided large intestinal structures, wall appearance, and motility
7 Thoracic	Lung, pleura	Pleural surface, pleural fluid, and pulmonary abnormalities

### Data preparation and preprocessing

The preprocessing and data management pipeline was gradually refined during exploratory model development. Based on these preliminary analyses, image preprocessing, quality control, and dataset partitioning procedures were optimized before training the final model.

**Fig. 2:**
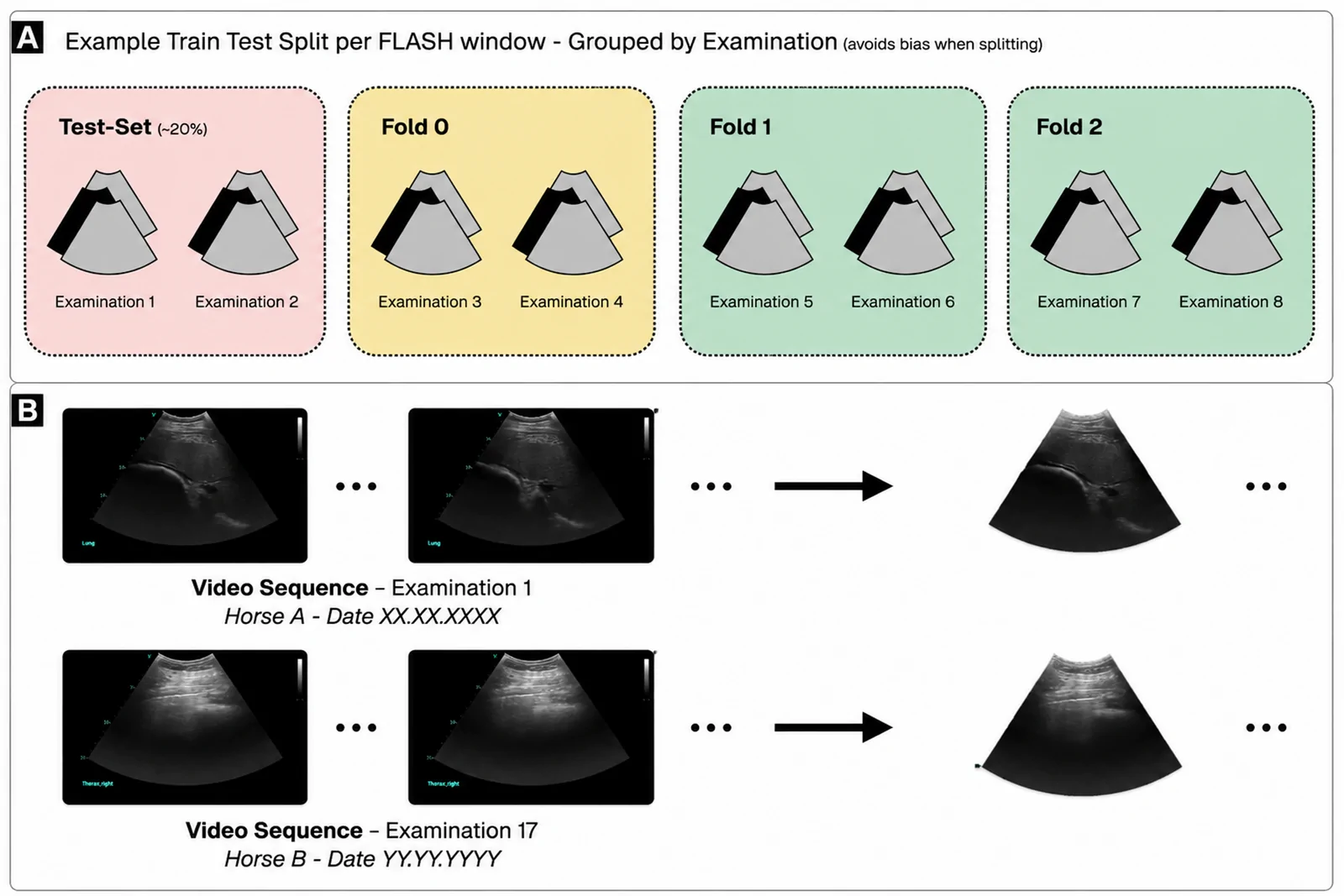
**A** Where multiple examinations from the same horse were used in the dataset, images originating from the same examination were always assigned to a single dataset partition to reduce bias. **B** Preprocessing workflow. Ultrasound video sequences were decomposed into individual frames. Textual annotations and image regions outside the ultrasound sector were removed, retaining only diagnostically relevant image content for subsequent model training

To prevent data leakage, dataset partitioning was performed at the examination level rather than at the image level. Each ultrasound examination consisted of a cine loop from which multiple image frames were extracted. Because these frames are highly similar and originate from the same acquisition, assigning them to different datasets could lead to overly optimistic performance estimates. Therefore, all frames extracted from a single examination were assigned to the same dataset partition (training, validation, or test) and were never split across different datasets. To maintain a balanced distribution of FLASH windows, examinations were grouped according to their acquisition date within each class before partitioning (Fig. 2A).

Ultrasound video sequences were decomposed into individual image frames. Each frame subsequently underwent preprocessing to remove textual annotations and other markings outside the ultrasound cone (Fig. 2B). The resulting images contained only the relevant ultrasound information and were stored with an alpha channel.

Image quality control was performed by the two board-certified examiners to remove empty frames and acquisition artifacts (e.g., completely black frames, frames without visible ultrasound information, severe motion artifacts, frames obtained during probe repositioning, or images with incomplete ultrasound sectors). Initially, frames were filtered using a black-level threshold. The resulting dataset was then manually reviewed, and remaining empty or corrupted images were removed. Exploratory analyses further demonstrated that FLASH windows 4 and 6 were substantially underrepresented and showed considerable anatomical overlap, resulting in consistently poor classification performance. Because reliable classification of these windows could not be achieved despite repeated optimization, they were excluded from the final model development and evaluation. From an initial dataset of 23,454 images (FLASH windows 1, 2, 3, 5, and 7), 211 images were removed automatically during quality filtering and a further 3,243 images were excluded following manual review, leaving 20,000 images (85.27% of the initial dataset). To avoid overrepresentation of individual ultrasound examinations, a maximum of 99 frames per ultrasound examination (cine loop) was retained. This resulted in the final training, validation, and test datasets described in Sect. "Dataset composition".

### Dataset partitioning and model training

The complete dataset was first divided into an independent test set comprising approximately 20% of all examinations. The remaining data were used for model development and hyperparameter optimization.

A three-fold cross-validation strategy was employed. The development dataset was partitioned into three folds at the examination level. During each cross-validation run, one-fold was used for validation while the remaining two folds were used for training. This process was repeated three times so that each fold served once as the validation set.

Hyperparameter optimization was performed using Optuna [60, 61]. For each trial, a model was trained using three-fold cross-validation, and the mean validation accuracy across all folds was used as the optimization objective. Ten Optuna trials were conducted to evaluate different hyperparameter configurations.

After hyperparameter optimization, the best-performing configuration was selected. A final model was then trained using all data from the three development folds combined. Model performance was subsequently assessed on the independent test set, which had not been used during training, validation, or hyperparameter optimization.

### Image transformations and augmentation

Several data augmentation strategies were evaluated during preliminary experiments, including random horizontal flipping, color jittering, affine transformations, and perspective transformations. However, these augmentations did not improve model performance and were therefore omitted from the final training pipeline.

The final preprocessing pipeline consisted of converting images with an alpha channel into RGB images using a black background, followed by center cropping, and resizing to the input dimensions required by the network architecture (Fig. 2B). Finally, images were normalized using the ImageNet mean and standard deviation values, consistent with the pretraining procedure of the EfficientNet-B0 architecture.

To reduce overfitting, regularization was applied through dropout and weight decay during model training.

## Results

### Dataset composition

After exclusion of the underrepresented and morphologically similar classes FLASH 4 and FLASH 6, the final dataset comprised five ultrasound window classes: ventral (FLASH 1), gastric (FLASH 2), nephrosplenic (FLASH 3), duodenal (FLASH 5), and thoracic (FLASH 7). Dataset partitioning was performed at the examination level to prevent data leakage between training, validation, and test sets. Multiple examinations from the same horse could be present in the dataset; however, images originating from the same examination were always assigned to a single dataset partition.

To reduce potential overfitting to individual examinations, the number of training samples per examination was limited to a maximum of 99 frames. Following balancing procedures, the training dataset comprising 2,437 images showed a near-uniform class distribution, with class frequencies ranging from 16.3% (FLASH window 1) to 22.7% (FLASH window 2). The independent test set contained 6,119 images, with a naturally imbalanced class distribution reflecting the available data (Table 2).

**Table 2:** Class distribution of the training and test datasets. The table presents the absolute number (*n*) and relative proportion (%) of ultrasound images for each FLASH window in the training and test datasets. The training dataset was balanced through data augmentation, whereas the test dataset retained its original class distribution

Class	Train (*n*)	Train (%)	Test (*n*)	Test (%)
FLASH 1	396	16.25	513	8.38
FLASH 2	553	22.69	1175	19.20
FLASH 3	498	20.43	862	14,09
FLASH 5	495	20.31	2176	35.56
FLASH 7	495	20.31	1393	22.77
Total	2437	100.00	6119	100.00

### Classification performance

Using EfficientNet-B0 as the backbone architecture, the final model achieved an overall test accuracy of 80.8% on the independent test dataset.

Performance varied between ultrasound windows (Table 3). The highest class-specific performance was observed for the duodenal window (FLASH 5), which achieved perfect recall (1.00), a precision of 0.884, and an F1-score of 0.938. The nephrosplenic window (FLASH 3) also demonstrated strong performance, with a precision of 0.965 and an F1-score of 0.852.

**Table 3:** Classification performance of the different FLASH windows was assessed using precision, recall, and F1-score. Precision reflects the reliability of positive predictions, recall measures the ability to identify all true instances of a class, and the F1-score summarizes both metrics into a single measure by calculating their harmonic mean. Macro-averaged metrics assign equal importance to all classes regardless of their size, whereas weighted averages account for class imbalance by weighting each class according to its number of samples

Class (window)	Precision	Recall	F1-score	Support
FLASH 1	0.407	0.844	0.549	513
FLASH 2	0.820	0.781	0.800	1175
FLASH 3	0.965	0.763	0.852	862
FLASH 5	0.884	1.000	0.938	2176
FLASH 7	0.962	0.546	0.697	1393
Macro average	0.808	0.787	0.767	6
Weighted average	0.861	0.808	0.812	6119

The gastric window (FLASH 2) achieved balanced classification performance, with a precision of 0.820, recall of 0.781, and F1-score of 0.800.

Lower performance was observed for the ventral (FLASH 1) and thoracic (FLASH 7) windows. The ventral window achieved high recall (0.844) but comparatively low precision (0.407), indicating frequent false-positive classifications. In contrast, the thoracic window showed high precision (0.962) but lower recall (0.546), suggesting that a substantial proportion of thoracic images were misclassified as other classes (Table 3).

Overall, the model achieved a macro-averaged precision, recall, and F1-score of 0.808, 0.787, and 0.767, respectively. The weighted averages were 0.861 for precision, 0.808 for recall, and 0.812 for F1-score, indicating robust classification performance despite class imbalance in the independent test dataset.

### Confusion matrix

**Fig. 3:**
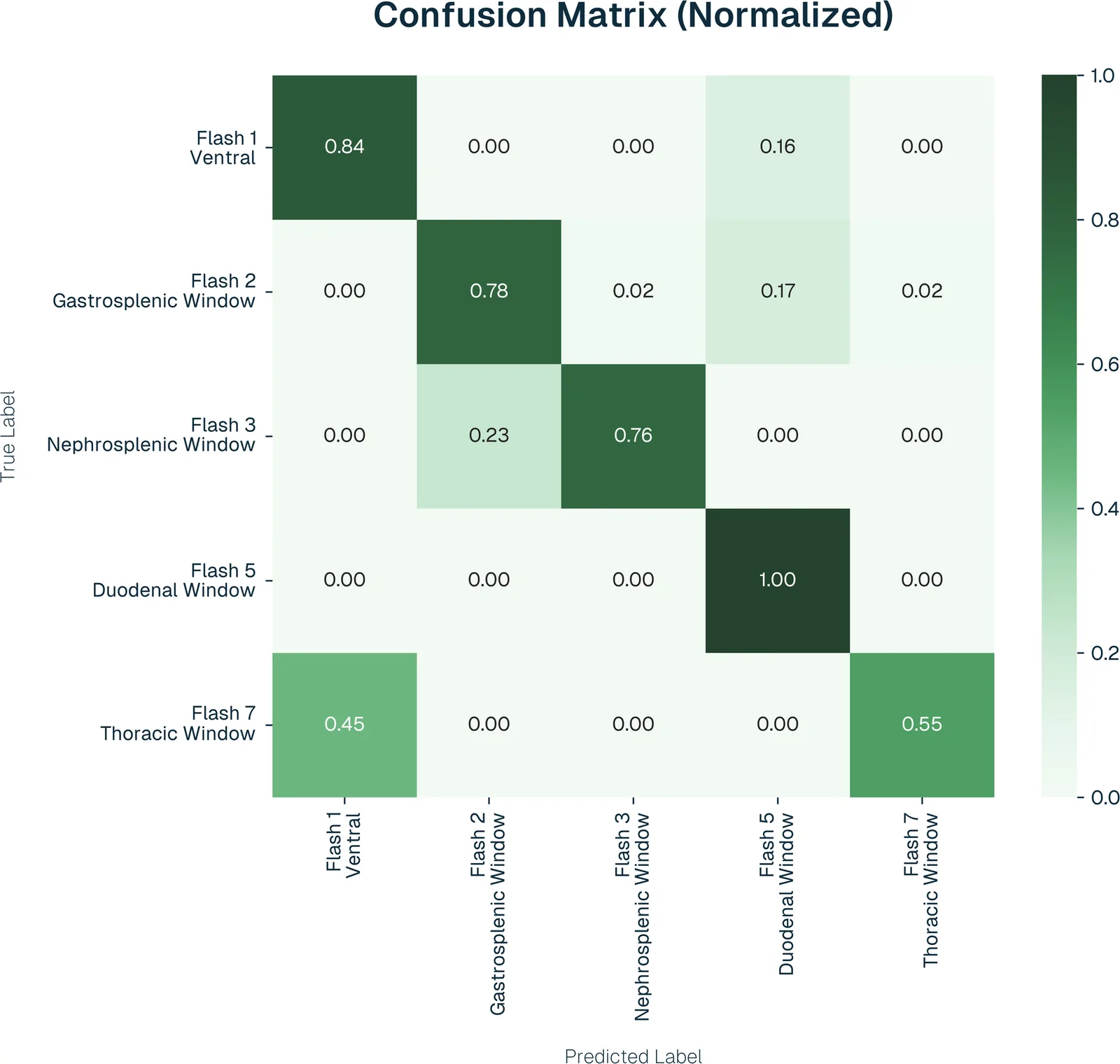
Normalized confusion matrix of the classification model across the five ultrasound windows. Diagonal values indicate correctly classified samples, whereas off-diagonal values represent misclassifications. The highest classification accuracy was achieved for FLASH 5 (100%), while FLASH 7 showed the lowest accuracy (55%) and was most frequently misclassified as FLASH 1 (45%)

Analysis of the confusion matrix revealed distinct patterns of misclassification between specific ultrasound windows (Fig. 3). The duodenal window (FLASH 5) showed the strongest performance, with all test images correctly classified, corresponding to a recall of 1.00. Similarly, the nephrosplenic window (FLASH 3) demonstrated high classification accuracy, although approximately 23% of images were misclassified as the gastric window (FLASH 2). While 84% of ventral images (FLASH 1) were correctly classified, 16% were predicted as the duodenal window (FLASH 5).

**Fig. 4:**
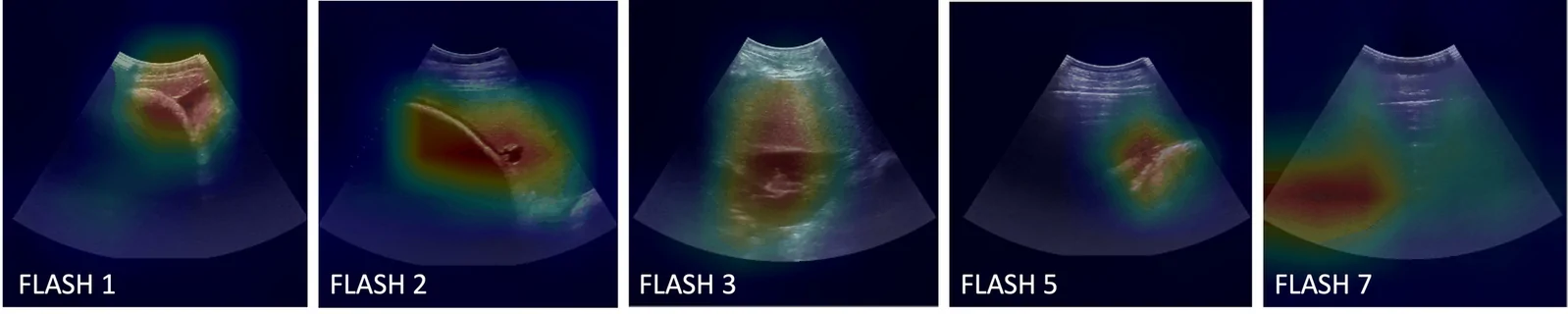
Attention heatmaps (Grad CAM) for different FLASH acquisitions. The model consistently focuses on relevant anatomical structures in FLASH 1, 2, 3, and 5. In contrast, for FLASH 7 the attention is predominantly directed toward the image border rather than the underlying anatomy in some images

The most pronounced confusion was observed between the ventral (FLASH 1) and thoracic (FLASH 7) windows, with only 55% of thoracic images correctly identified, and 45% misclassified as the ventral window (FLASH 1). This finding is consistent with the lower recall observed for FLASH 7. When evaluating attention heat maps, there was a visible shift of attention toward the image border in some of the FLASH 7 images (Fig. 4).

A second source of confusion was observed between the gastric (FLASH 2) and nephrosplenic (FLASH 3) windows. Approximately 23% of nephrosplenic images were classified as gastric, while only 2% of gastric images were classified as nephrosplenic. This asymmetric confusion may reflect differences in image variability between the two anatomical regions.

**Fig. 5:**
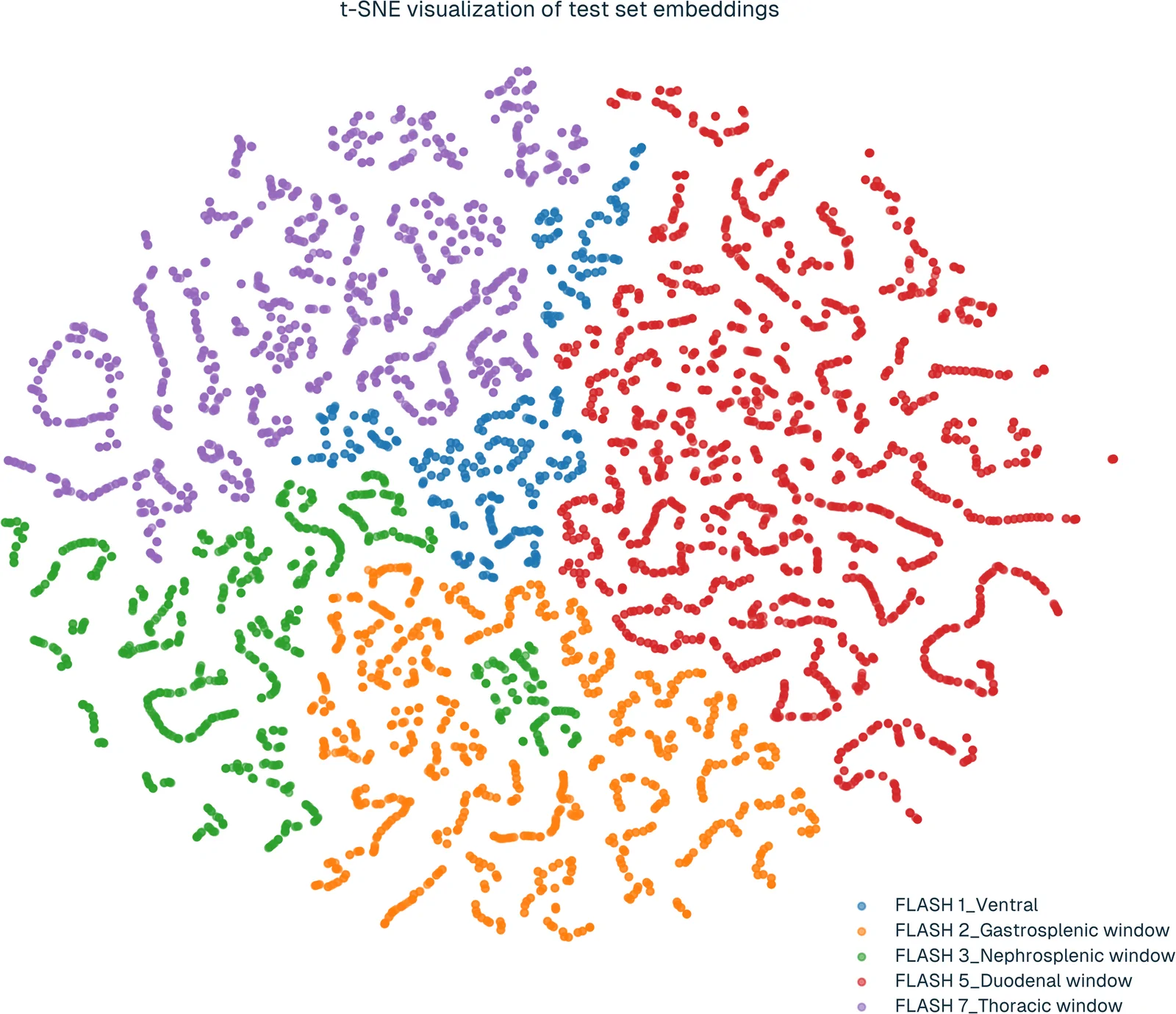
Two-dimensional t-SNE visualization of feature embeddings extracted from the test set. Each point corresponds to an individual ultrasound image and is colored by its FLASH window label. The observed clustering suggests that the learned embeddings capture characteristics associated with the different anatomical windows

Overall, misclassifications were concentrated between anatomically related and adjacent ultrasound windows, whereas confusion between more distinct regions was uncommon. The absence of substantial confusion involving the duodenal window further indicates that this view contains characteristic image features that can be reliably recognized by the model. The t-SNE dimensionality reduction demonstrated class-specific clustering of the learned embeddings, with moderate overlap between some FLASH windows (Fig. 5).

## Discussion

This study shows the potential capability of a deep learning tool to distinguish between the different FLASH windows in healthy horses.

Classification of FLASH windows proved challenging because the protocol defines anatomical regions rather than discrete organ compartments. Adjacent windows frequently contain overlapping structures due to the large size and mobility of the equine gastrointestinal tract. In healthy horses, ultrasonographic appearances are often highly similar between neighboring windows, particularly when predominantly large intestinal structures are visualized as in FLASH windows 4 and 6 [62]. Previous studies have demonstrated that differentiation between specific regions of the equine large colon is inherently difficult in healthy horses, as reliable ultrasonographic landmarks are often absent and sonographic appearances overlap considerably [63]. In contrast, pathological conditions may generate more distinctive regional features, such as intestinal distension, altered wall thickness, or reduced motility, which could facilitate anatomical localization [64]. Consequently, the clinical relevance of the FLASH protocol primarily emerges in presence of pathological findings, where regional differences become diagnostically meaningful. While healthy examinations may show overlapping sonographic appearances between adjacent windows, disease-associated changes often generate window-specific abnormalities that support both localization and clinical interpretation. This characteristic distinguishes ultrasonography from other diagnostic imaging modalities, as its diagnostic accuracy and interpretability are substantially more dependent on the examiner’s expertise and technique than modalities such as radiography or computed tomography.

In contrast to human point-of-care ultrasound protocols (e.g., Bed Lung Ultrasound in Emergency – BLUE; Focused Assessment with Sonography in Trauma - FAST) [65, 66], which are based on strictly defined acoustic windows and reproducible scanning positions, equine abdominal ultrasound is characterized by a more flexible scanning approach driven by large, mobile abdominal organs and less fixed anatomical landmarks (vs. human liver/spleen boundaries) [67–69]. Furthermore, the exact position of the probe during image acquisition may vary slightly between operators, potentially contributing to transitional images that share characteristics of multiple windows. Some images may therefore represent anatomical “border zones” between neighboring FLASH windows rather than perfectly distinct categories [2]. Studies have shown distinct inter- and intraobserver variability in equine ultrasound even in standardized structures [70].

Notably, nearly half of all FLASH 7 images were classified as FLASH 1, indicating a pronounced overlap between these categories. This finding suggests that the visual distinction between thoracic and ventral windows is less dependent on individual anatomical structures than on their spatial context during the examination. When pleural interfaces or lung artifacts are incompletely visualized, thoracic images may resemble ventral abdominal views and become difficult to distinguish. Because the model was trained on isolated frames rather than video sequences, it could not utilize dynamic information such as respiratory motion, probe movement, or the gradual transition between anatomical regions. In such situations, ultrasound images may instead be dominated by non-specific soft tissue patterns, reverberation artifacts, or gas interfaces, thereby reducing the availability of discriminative anatomical features for both human observers and AI-based classification systems. Consequently, images lacking clearly visible pleural landmarks may have been classified according to more general image characteristics shared with ventral abdominal views. Therefore, the Grad-CAM heat maps pattern observed for FLASH 7 suggests a potential reliance on non-anatomical image features, which may have contributed to the reduced interpretability of the model’s predictions.

The observed classification ambiguity may also reflect the dynamic nature of FLASH examinations. Ultrasound examinations are performed as continuous scanning procedures rather than as collections of isolated static images. Consequently, individual frames may capture transitional probe positions between adjacent windows and contain mixed anatomical information. This effect is likely amplified by gas-related artifacts, variable organ visibility, and the intermittent appearance of key landmarks such as the duodenum or pleural interfaces. The confusion observed between windows 1 and 7 may additionally reflect the anatomical continuity of the thoracoabdominal junction, where thoracic and abdominal structures can appear simultaneously within the ultrasound field.

Equine ultrasonography is highly operator-dependent [3]. Probe position, angulation, pressure, image settings, and individual scanning technique can substantially influence image appearance. Even when standardized FLASH protocols are followed, subtle differences in image acquisition between operators may substantially alter the sonographic appearance of a given examination site. In addition, anatomical variability related to body conformation, intestinal filling, and patient movement may further contribute to heterogeneous image characteristics within the same FLASH category. Such variability increases intra-class heterogeneity and may reduce the ability of the model to identify stable and reproducible feature representations. Furthermore, the model may have partially relied on acquisition-related image characteristics rather than exclusively on anatomical landmarks, a phenomenon known as shortcut learning [71].

A central limitation of the present study concerns the definition of ground truth. FLASH windows are operator-defined examination regions with partially overlapping anatomical content rather than strictly separated anatomical compartments. Consequently, some degree of annotation uncertainty is unavoidable, particularly in frames acquired during transitions between adjacent scanning positions. This intrinsic ambiguity likely places an upper limit on achievable classification performance independent of model architecture.

Another limitation was the imbalance of the dataset. Windows 4 and 6 (left and right middle third) were substantially underrepresented, which likely contributed to their poor classification performance and ultimately their exclusion from the final model. Furthermore, the comparatively lower classification performance between windows 4 and 6 may be explained also by anatomical and not only dataset-related factors. In healthy horses, these FLASH windows predominantly display large intestinal structures with similar ultrasonographic appearance, limiting the availability of distinctive landmarks. Future studies should include larger and more balanced datasets collected from multiple operators and institutions to improve robustness and generalizability.

A further limitation is that the dataset was derived from only six horses. Although data leakage at the examination level was prevented by ensuring that all frames from a single examination remained within the same dataset partition, different examinations from the same horse could be included in both the training and test datasets. Consequently, the model may have learned horse-specific image characteristics in addition to generalizable anatomical features, potentially leading to an overestimation of its performance. Future studies should validate the model using an independent dataset from previously unseen horses.

Although the present study focused on automated FLASH window recognition, the clinical value of such systems may extend beyond classification accuracy. AI-assisted tools could support anatomical orientation, improve examination standardization, and assist less experienced operators during image acquisition. In addition to its diagnostic applications, AI-assisted FLASH recognition may have educational value by supporting novice examiners during training and standardizing image acquisition. Such systems could facilitate earlier acquisition of practical ultrasonographic skills using annotated image datasets and supervised AI feedback, potentially decreasing the number of repeated training examinations required in live horses and aligning with the principles of refinement and reduction within the 3Rs framework (Replacement, Reduction, and Refinement) [72–75]. In veterinary education, AI-assisted ultrasound systems may support this goal by enabling structured, simulation-based learning and reducing the need for repeated examinations on live horses which place a considerable burden on teaching horses, while client-owned horses are often unavailable or unsuitable for prolonged training sessions. In future, AI tools may serve as supplements to conventional teaching, allowing students to acquire basic scanning orientation and familiarity with standardized FLASH windows before hands-on training, thereby reducing unnecessary scanning time on teaching horses while making practical sessions more efficient. This may enable more efficient use of practical teaching time by allowing students to identify the relevant anatomical windows more quickly once they perform examinations on live animals. The proposed AI system should be interpreted as a decision-support tool rather than a replacement for clinical expertise. Particularly in the context of equine colic diagnostics, ultrasound findings must always be integrated with clinical examination and patient history. The primary role of the algorithm is therefore to assist in the standardization and interpretation of FLASH window acquisition, especially for less experienced operators, rather than to provide autonomous diagnostic conclusions.

Future steps are to develop a virtual reality ultrasound dummy to help students in skills labs and inexperienced veterinarians in the field to increase their ultrasound skills and gain experience without the need to practice on live horses. Hereby, the tool may help increase equine welfare by reducing the need of live teaching horses - thus meeting the criteria of the 3 R´s (Replace, Reduce, Refine)-, and by providing more advanced skills as a first-day competency.

## Conclusions

This study demonstrates the feasibility of applying deep learning to the recognition of standardized equine FLASH ultrasound windows. Although classification performance varied considerably between individual windows and is not yet sufficient for routine clinical or educational use, the model successfully identified several FLASH windows and consistently focused on anatomically relevant image regions. These findings provide a proof-of-concept for AI-assisted ultrasound image recognition in horses. Future studies should include larger and more diverse datasets, external validation, and video-based analysis to improve robustness and better reflect the dynamic nature of clinical ultrasonography. In addition, incorporating organ-level annotations and probe position information during training may further enhance discrimination between anatomically similar FLASH windows.

## Data Availability

The datasets generated and/or analysed during the current study are available from the corresponding author on reasonable request.

## Abbreviations

FLASH: Fast Localised Abdominal Sonography of Horses
